# The effect of lipid metabolism disorder on patients with hyperuricemia using Multi-Omics analysis

**DOI:** 10.1038/s41598-023-45564-8

**Published:** 2023-10-24

**Authors:** Lili Ma, Jing Wang, Li Ma, Yan Ge, Xian Min Wang

**Affiliations:** 1 Department of Internal Medicine, Shengzhou Hospital of Traditional Chinese Medicine, Shaoxing, 312400 China; 2 Xinjiang Laboratory of Respiratory Disease Research, Hospital of Traditional Chinese Medicine Affiliated to Xinjiang Medical University, Urumqi, 830000 China; 3 Department of Endocrinology, Affiliated Hospital of Traditional Chinese Medicine, Xinjiang Medical University, Urumqi, 830000 China; 4 Department of Internal Medicine, Changji Hospital of Traditional Chinese Medicine, Changji, 831100 China; 5 Department of Scientific Research Management, Affiliated Hospital of Traditional Chinese Medicine, Xinjiang Medical University, Urumqi, 830000 China

**Keywords:** Diseases, Medical research

## Abstract

A multiomics study was conducted to investigate how lipid metabolism disorders affect the immune system in Xinjiang patients with hyperuricemia. The serum of 60 healthy individuals and 60 patients with hyperuricemia was collected. This study used LC–MS and HPLC to analyze differential lipid metabolites and enrichment pathways. It measured levels of immune factors tumor necrosis factor-α (TNF-α), interleukin 6 (IL-6), carnitine palmitoyltransferase-1 (CPT1), transforming growth factor-β1 (TGF-β1), glucose (Glu), lactic acid (LD), interleukin 10 (IL-10), and selenoprotein 1 (SEP1) using ELISA, as well as to confirm dysregulation of lipid metabolism in hyperuricemia. 33 differential lipid metabolites were significantly upregulated in patients with hyperuricemia. These lipid metabolites were involved in arachidonic acid metabolism, glycerophospholipid metabolism, linoleic acid metabolism, glycosylphosphatidylinositol (GPI)—anchor biosynthesis, and alpha-Linolenic acid metabolism pathways. Moreover, IL-10, CPT1, IL-6, SEP1, TGF-β1, Glu, TNF-α, and LD were associated with glycerophospholipid metabolism. In patients with hyperuricemia of Han and Uyghur nationalities, along with healthy individuals, significant differences in CPT1, TGF-β1, Glu, and LD were demonstrated by ELISA (*P* < 0.05). Furthermore, the levels of SEP1, IL-6, TGF-β1, Glu, and LD differed considerably between groups of the same ethnicity (*P* < 0.05). It was found that 33 kinds of lipid metabolites were significantly different in patients with hyperuricemia, which mainly involved 5 metabolic pathways. According to the results of further studies, it is speculated that CPT1, TGF-β1, SEP1, IL-6, Glu and LD may increase fatty acid oxidation and mitochondrial oxidative phosphorylation in patients through glycerophospholipid pathway, reduce the rate of glycolysis, and other pathways to change metabolic patterns, promote different cellular functions, and thus affect the disease progression in patients with hyperuricemia.

## Introduction

Hyperuricemia (HUA) is resulted from reduced uric acid excretion or abnormal purine metabolism and is inextricably linked to chronic kidney disease^1^, metabolic syndrome^2^, and type 2 diabetes^3^. A study found that HUA prevalence is 13.5% (17.3% in men, 10.0% in women) when age and sex are standardized^3^. Uric acid (UA) is the only diagnostic indicator of hyperuricemia and is only produced by the xanthine oxidoreductase (XOR) oxidation of xanthine and hypoxanthine^4^. Furthermore, adipocytes produce uric acid through XOR^5^_,_ indicating that adipose tissue may be another vital source of uric acid. Researchers have revealed that metabolic disorders, oxidative stress, and renin-angiotensin system dysfunction contribute to HUA progression^6–9^. Therefore, hyperuricemia may induce disorders of lipid metabolism.

By utilizing mass spectrometry-based metabolic approaches, it is possible to profile cell metabolism directly and uncover the mechanisms of metabolic pathway transcriptional activation^9,10^. Metabolism and lipidomics can be efficiently combined to study changes in lipids during various biological processes, clarify the processes and mechanisms that underlie related biological activities, and identify markers in different organisms^11^. A recent study found that potential differential metabolites of numerous lipids are associated with HUA and participate in lipid metabolism pathways, such as glycerophospholipid metabolism, sphingolipid metabolism, and glycosylphosphatidylinositol (GPI)-anchor biosynthesis^12^. Moreover, the CXC ligand 13 (CXCL-13) pathway can induce lipid metabolism disorder in hyperuricemia^12^. Chicory, plantaginis semen, and dendrobium officinalis may reduce uric acid levels by regulating lipid metabolism^14–16^. Studies on lipid metabolism have focused primarily on screening biomarkers associated with HUA and their metabolic pathways. The underlying molecular mechanisms by which hyperuricemia leads to lipid metabolism disorders remain unclear.

It is hypothesized that immune factors TNF-α, CPT1, IL-6, SEP1, TGF-β1, IL-10, Glu and LD are involved in the regulatory pathway of lipid metabolism. The regulation of metabolic homeostasis by these signaling molecules may be participated in the HUA's pathogenesis. Now, UPLC and LC–MS were used to analyze lipid metabolism in patients with hyperuricemia, identify different lipid metabolites, and clarify lipid-related metabolic pathways. At the same time, ELISA was utilized to measure the changes of the important regulatory components IL-10, CPT1, TNF-α, SEP1, TGF-β1, IL-6, Glu and LD levels. The study aims to identify potential targets for further intervention in HUA.

## Materials and methods

### Baseline information

120 persons participated in this cross-sectional study, 60 patients with hyperuricemia were treated between January 2021 and December 2022 to the Xinjiang Uyghur Autonomous Region Hospital of Traditional Chinese Medicine. The hospital’s physical examination center recruited 60 healthy controls of matched age and gender. The Ethics Committee of the Xinjiang Uyghur Autonomous Region Hospital of Traditional Chinese Medicine (No.20XE0115-1) approved the study, which was conducted with the informed consent of all participants. All programs related to human participants were developed according to the Declaration of Helsinki (updated in 2013)^18^.

### Inclusion and exclusion criteria

Participant inclusion criteria included a 30–70-year-old age range and living more than three generations in Xinjiang. Patients with cardiovascular and cerebrovascular diseases, severe liver dysfunction, severe nephropathy, tumors, and psychosis were excluded.

### Hyperuricemia diagnosis

Based on the Guidelines for Diagnosis and Treatment of Hyperuricaemia and gout in China (2019)^19^: Over 420 mol/L of uric acid is considered hyperuricemia.

### Samples collection of serum

A fast of 12 h was followed by collection of 5 mL of venous blood, followed by separation of serum. UA concentrations were measured using an enzyme uricase method. This study used a Mindray Co, Shenzhen, China, automatic biochemical analyzer to measure high-density lipoprotein cholesterol (HDL-C), fasting blood glucose (FBG), Very Low-Density Lipoprotein Cholesterol (VLDL-c), creatinine (CR), blood urea nitrogen (BUN), triglycerides (TG), low-density lipoprotein cholesterol (LDL-C), as well as total cholesterol (TC) levels.We stored the total serum samples at − 80 °C before performing the experiments.

### Lipid extraction

Sodium heparin blood collection was used to collect 5 mL of whole blood, which was immediately inverted to ensure homogenization. Using a low temperature and elevated speed centrifuge (5430 R, Eppendorf), we centrifuged whole blood for 10 min at − 1 °C using 3000 rpm at 3000 rpm after plasma was stored at − 80 °C. 240 L of precooled methanol and 200 L of water were vortexed with 100 L of plasma. For 20 min, 800 L of MTBE were vortexed and sonicated in a water bath at low temperatures. Following 30 min at room temperature and incubation at 10 °C, samples were centrifuged for 15 min at 14,000 g. Nitrogen was used as a drying agent for the organic phase. A mixture of 200 L of 90% Isopropanol/acetonitrile was mixed with it and centrifuged at 14,000 g for 15 min at 10 degrees Celsius. Lastly, mass spectrometric analysis was performed on the supernatants. We took it by a positive/negative ion fingerprints analysis, respectively. Extracts from all samples were mixed in equal volumes, and QC samples were prepared with the same sample volume. A − 20 °C refrigerator was used to store the extraction reagents.

### Analyses using LC–MS

Lipids were separated using an HPLC system (Thermo Scientific). The chromatographic analysis was conducted with a 3 L injection volume, a 45 °C column temperature, and a flow rate of 300 L per minute.

During phase A (ACN/H2O [6:4 v/v]), 10 mM ammonium formate and acetonitrile solutions were combined. As mobile phase B, ACN:IPA (2:9 v/v) was used with 10 mM isopropyl ammonium formate. The chromatography gradient started at 30% mobile from 0 to 2 min phase B, increasing to 100% from 2 to 25 min; mobile phase B was then left at 30% from 25 to 35 min. Throughout the entire analysis, temperatures of 10 °C were maintained in an autosampler and analyzed in random order using continuous analysis. An electrospray ionization mass spectrometer with Q active plus was used to perform untargeted lipidomic analyses. According to the source conditions, the heater temperature was 300 °C, the sheath gas flow rate was 45 ARB, the auxiliary gas flow rate was 15 ARB, the sweep gas flow rate was 1 ARB, the spray voltage was 3.0 kV, the capillary temperature was 350 °C, and the s-lens rf level was 50%. There was a scanning range of 200–1800 for MS1. Negative: The heater tempo was 300 °C, sheath gas flow rate was 45 ARB, aux gas flow rate was 15 ARB, sweep gas flow rate was 1 ARB, spray voltage was 2.5 kV, the sweep gas flow rate was 1 ARB, the spray voltage of 2.5 kV, the capillary tempo was 350 °C, and the s-lens rf level was 60%. After each full scan (full scan), 10 fragment profiles (MS2, HCD) were collected to determine the mass-to-charge ratios of lipid molecules and fragments. As of M/Z 200, 70,000 pixels were used in MS1, while MS2 had a resolution of 17,500. We purchased chemical components from Thermo including ethylene, isopropyl alcohol, and methanol. In addition to the ammonium formate, a spectrograph (Q-Exactive Plus from Thermo Scientific) and a chromatography column (ACQUITY UPLC CSH C18 from Waters) were purchased. All chemicals and solvents were analytically pure or chromatographic grade.

### ELISA analysis

Serum samples were read from plates using a Versa Max microplate reader (Bio-Rad, Hercules, CA, USA). Then, using SoftMax pro 6.2.2 measured the levels of interleukin 6 (IL-6), carnitine palmitoyltransferase-1 (CPT1), selenoprotein 1 (SEP1), tumor necrosis factor-α (TNF-α), lactic acid (LD), interleukin 10 (IL-10), glucose (Glu) and transforming growth factor-β 1 (TGF-β1). The contents of these factors were calculated using the following formulas. Pattern recognition was done using SIMCA-P 14.1, an application software package.

### Data processing

LipidSearch data were normalized by removing any molecules with missing values over 50% during data extraction. The research treated the data with Pareto-scale before performing pattern recognition with SIMCA-P 14.1 which was from Umetrics in Umea, Sweden. The data analysis was conducted using partial least squares discriminant analysis (PLS-DA), orthogonal partial least squares discriminant analysis (OPLS-DA), and principal component analysis (PCA). Using seven cycles of linear regression interactions, R^2^Y and Q^2^ were determined. There was a t-test as well as a fold-shift analysis included in the univariate statistical analysis. R software created a volcano plot, hierarchical clustering analysis, bubble map, and correlation analysis. For path enrichment analysis, KEGG(www.kegg.jp/kegg/kegg1.html) was used.

### Other statistical analyses

Statistics were analyzed and processed using SPSS statistics (Version 25) (Chicago, IL, USA). Normally distributed measurements were expressed as normal data. In both comparisons, t tests were used for two groups of measurement data, and comparisons between multiple groups were based on analysis of variance. A X^2^ test was used for comparison of counts. Correlations were analyzed using Pearson’s correlation. Each test was statistically significant if *P* < 0.05 or *P* < 0.001. Logistic regression results are expressed as exp (B) (OR value) and 95%CI.

### Ethics approval

Ethics Committee of the Hospital of Traditional Chinese Medicine, Xinjiang Uygur Autonomous Region, has approved the project (No. 20XE0115-1).

## Results

### Characteristics of the demographic and clinical sample

Table 1 displays the Characteristics of the demographic and clinical sample features of 120 individuals (74 males and 46 females) who were included in this study. Among the patients with hyperuricemia, the weight, BMI, TG, TC, LDL-C, HDLC, VLDLC, BUN, Scr, SUA, and SUA/Scr were significantly higher than those of the healthy persons (*P* < 0.05 or *P* < 0.001).

**Table 1:** Characteristics of the two groups based on demographics and clinical data.

	Patients with hyperuricemia(N = 60)	Healthy persons(N = 60)	*P*-value
	Mean ± SD	Mean ± SD	
Age(years)	45.633 ± 9.288	43.417 ± 9.578	0.201
Weight (kg)	83.967 ± 15.119	66.667 ± 12.726	< 0.001
BMI (kg/m2)	27.56 ± 4.191	24.812 ± 4.01	< 0.001
TC (mmol/L)	4.598 ± 0.695	4.184 ± 0.572	< 0.001
TG (mmol/L)	2.309 ± 1.493	0.997 ± 0.355	< 0.001
HDLC (mmol/L)	0.924 ± 0.2	1.37 ± 0.295	< 0.001
LDLC (mmol/L)	3.141 ± 0.645	2.493 ± 0.497	< 0.001
VLDL-c (mmol/L)	0.533 ± 0.425**	0.322 ± 0.246	0.001
FBG (mmol/L)	4.765 ± 0.941	4.801 ± 0.488	0.794
BUN(mmol/L)	5.225 ± 0.977	4.64 ± 1.134	0.003
Scr(mmol/L)	85.267 ± 13.033	73.049 ± 12.527	< 0.001
SUA(mmol/L)	466.737 ± 27.467	287.169 ± 60.739	< 0.001
SUA/Scr	5.599 ± 0.901	3.96 ± 0.671	< 0.001

	N(%)	N(%)	*P*-value
Sex(%, man)	57(95.00)	17(28.3)	< 0.001
Ethnicity(%, Han ethnicity)	30(50.00)	30(50.00)	1.000

The sum of the mean x standard deviation (SD) or the percentage of individuals (%) is used to express data. UA is uric acid; FBG is fasting blood glucose; TG is triglycerides; BUN is blood urea nitrogen; LDL-C is low-density lipoprotein cholesterol; CR is creatinine; TC is total cholesterol; HDL-C is high-density lipoprotein cholesterol; BMI is body mass index; VLDL-c is Very Low-Density Lipoprotein Cholesterol.

### Logistic regression

Logistic regression was performed with Uric Acid Levels dependent variables. We added relevant variables to the regression model to build three regression models, as shown in the following Table 2. The criteria for inclusion were univariate meaningful variables and clinically known and recognized relevant variables. Model 1 is a rough model without any adjustment for confounders, model 2 is a statistically significant partially corrected model for univariate analysis, and Model 3 is a fully corrected model. The results showed that FBG, BUN, Scr, SUA/Scr migh affect the Uric Acid Levels.

**Table 2:** Logistic regression results of the association of participant characteristics with Uric Acid Levels.

Exposure	Model 1			Model 2			Model 3		
	t	B ( 95% CI)	*P* -value	t	B ( 95% CI)	*P* -value	t	B ( 95% CI)	*P* -value
Age(years)	–	–	–	–	–	–	1.126	0.134(− 0.102,0.37)	0.263
Sex	–	–	–	− 1.373	− 5.241(− 12.811,2.329)	0.173	− 1.241	− 4.786(− 12.433,2.861)	0.217
Ethnicity	–	–	–	–	–	–	− 0.089	− 0.219(− 5.128,4.689)	0.93
Weight (kg)	–	–	–	1.024	0.164(− 0.154,0.483)	0.308	1.282	0.214(− 0.117,0.546)	0.203
BMI(kg/m2)	–	–	–	− 0.752	− 0.387(− 1.406,0.633)	0.454	− 1.034	− 0.575(− 1.678,0.528)	0.303
TC (mmol/L)	–	–	–	0.897	1.647(− 1.993,5.287)	0.372	0.813	1.603(− 2.309,5.514)	0.418
TG (mmol/L)	–	–	–	0.614	0.992(− 2.212,4.195)	0.541	0.612	0.998(− 2.235,4.23)	0.542
HDLC (mmol/L)	–	–	–	− 0.857	− 4.114(− 13.627,5.398)	0.393	− 1.015	− 4.985(− 14.722,4.753)	0.312
LDLC (mmol/L)	–	–	–	–	–	–	–	–	–
VLDLC (mmol/L)	–	–	–	− 1.199	− 5.869(− 15.571,3.833)	0.233	− 1.277	− 6.347(− 16.201,3.507)	0.204
FBG (mmol/L)	–	–	–	− 2.722	− 3.625(− 6.265, − 0.984)	0.008	− 2.681	− 3.664(− 6.373, − 0.954)	0.009
BUN (mmol/L)	–	–	–	2.276	2.325(0.3,4.349)	0.025	2.043	2.14(0.063,4.218)	0.044
Scr (mmol/L)	–	–	–	34.034	4.067(3.83,4.304)	0	33.328	4.046(3.805,4.287)	0
SUA/Scr	–	–	–	52.005	71.295(68.577,74.013)	0	50.966	71.195(68.425,73.965)	0
Cardiovascular event	4.545	104.829(59.151,150.506)	0	0.986	2.995(− 3.03,9.019)	0.327	0.977	3.114(− 3.205,9.434)	0.331

### Non-targeted lipidomics analyses

#### QC results

**Figure 1:**
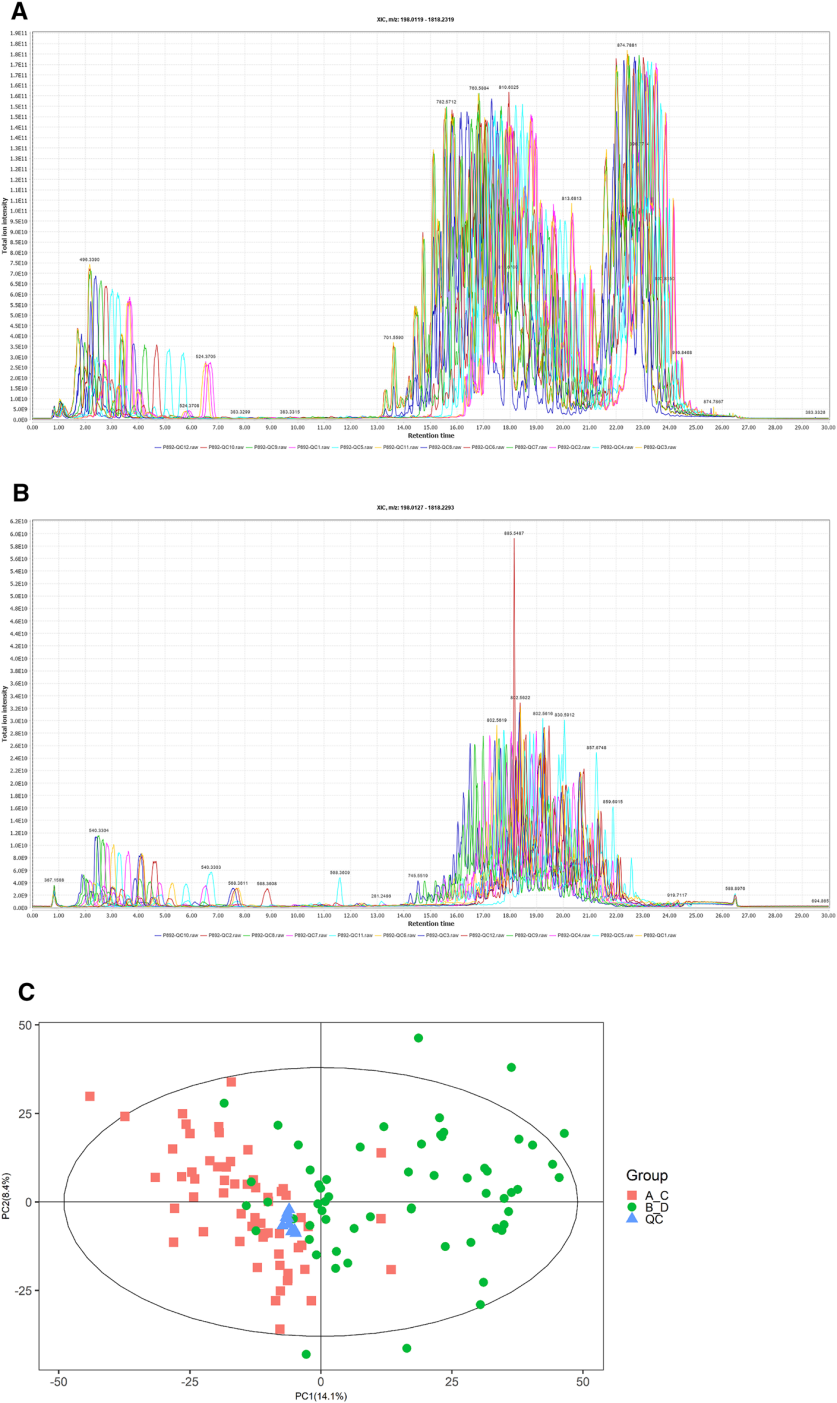
QC samples (**A**) Positive ion total ion flow diagram; **(B**) Negative ion entire ion flow diagram; (**C**) PCA score graphs for all samples.

In QC samples, the study detected various substance peaks, demonstrated in the total ion current diagram (TIC). A QC sample was characterized by positive and negative ions QC1 (Fig. 1A,B). The stability was excellent during the experiment (Fig. 1C). The PCA model diagram obtained through seven-fold cross-validation (seven cross-validation cycles) displays that there was a strong relationship between the clustering of QC samples and the metabolites in QC samples.

### An analysis of both qualitative and quantitative data

Qualitative and quantitative analyses were performed on the indicators collected from the ions in the positive and negative modes utilizing lipid search version 4.1 software. The research identified 1790 lipid metabolites, of which 913 were reduced, and 877 were increased (Annex in 1).

### Multivariate statistical analysis

**Figure 2:**
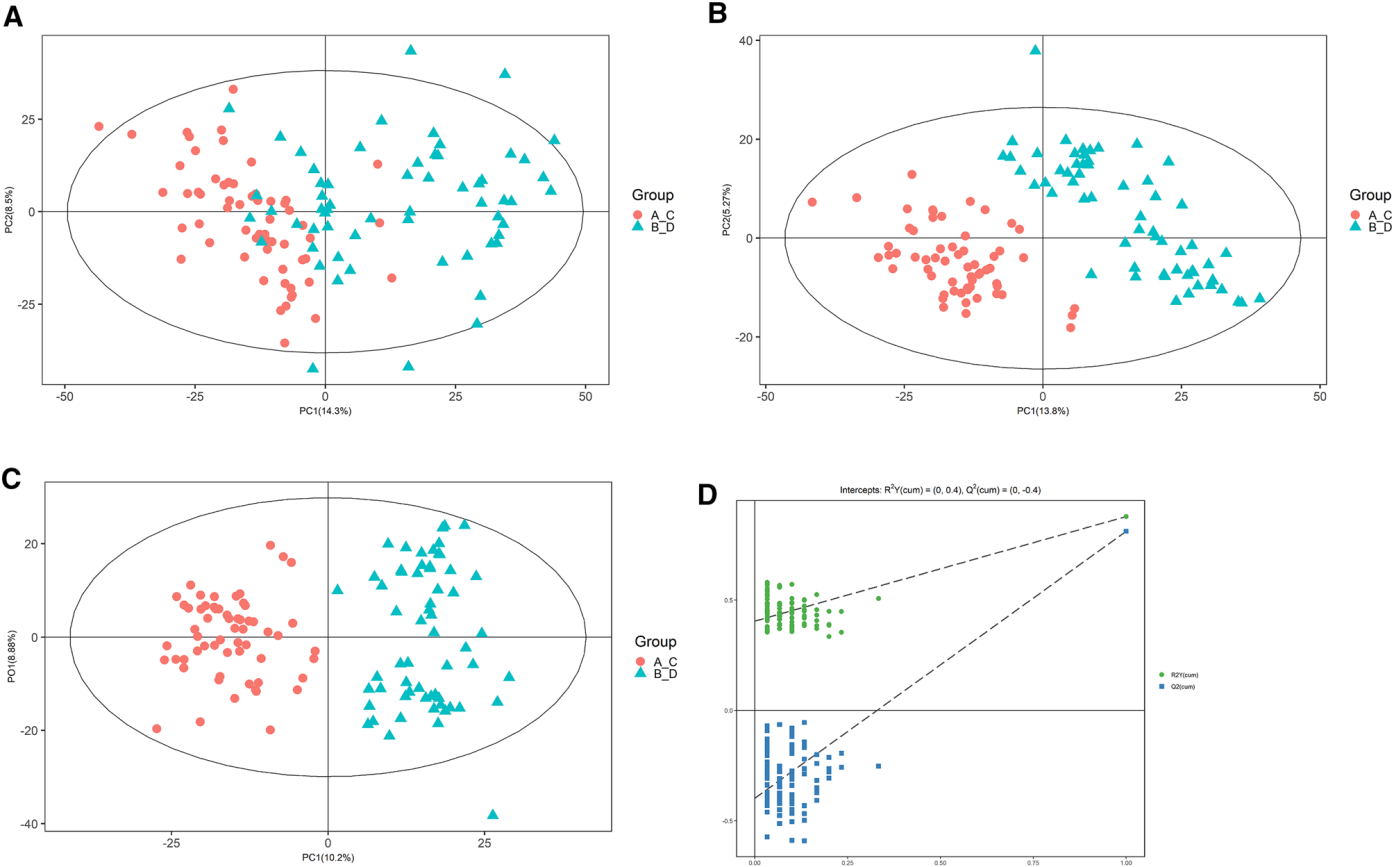
Two sets of score charts (**A**) Chart of PCA score; (**B**) Chart of PLS-DA scores; (**C**) Chart of OPLS-DA scores; (**D**) Test chart to replace OPLS-DA.

The PCA diagram demonstrated that most of the original data from both the patient and control groups were within the 95% confidence interval. A few discrete points demonstrated that the repeatability of the same group of samples increased during the detection process (Fig. 2A). The corresponding PLS-DA model displayed excellent quality and predictive ability (Fig. 2B). Plasma metabolites differed observably between patients and normals. To filter out noise irrelevant to classification, improve the model's analysis ability, and maximize the difference between different groups, the study modified PLS-DA by drawing an OPLS-DA score chart (Fig. 2C). Both groups scored significantly differently on OPLS-DA. The research found that when the projection (VIP) was > 1, the weights of the various variables were *P* < 0.05. The study used 7 cross-validation cycles (R^2^Y = 0.879, Q^2^ = 0.812) (Fig. 2D). According to the results, the model did not overfit but was stable.

### Lipid characteristics in HUA patients

**Figure 3:**
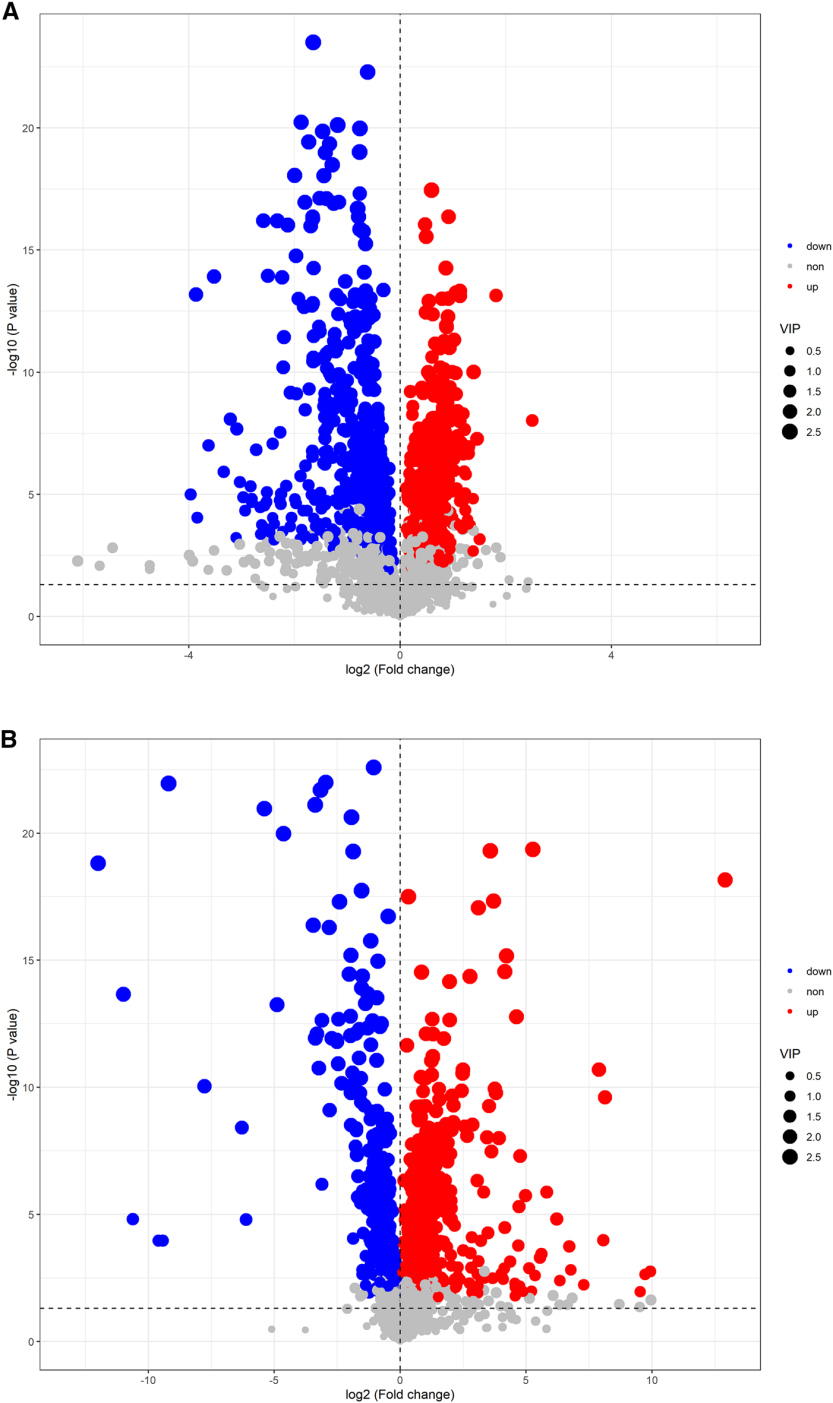

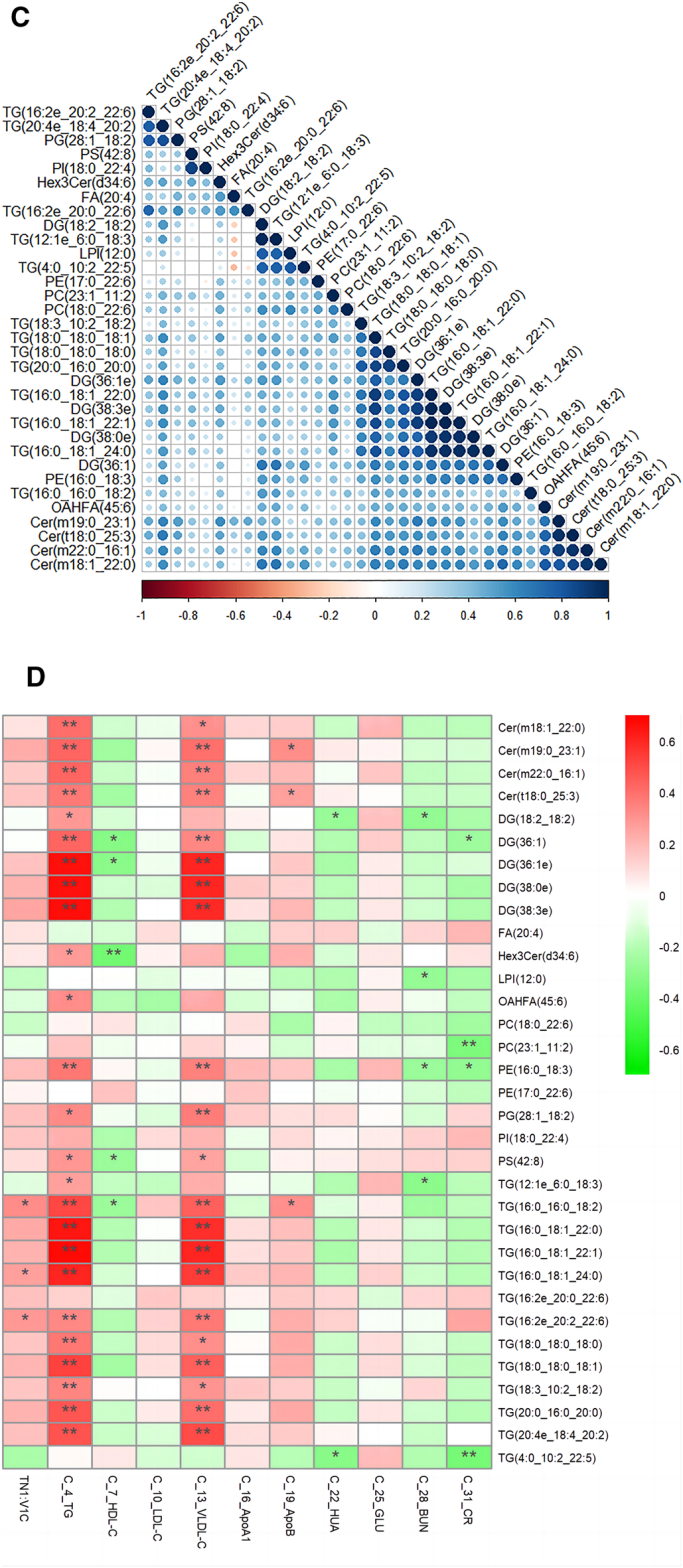
Differential lipid metabolites (**A**) Two groups of volcano maps; (**B**) HUA group of volcano map; (**C**) 33 differential lipid metabolites in HUA group of Clustering Heatmap; (**D**) Correlation analysis chart of clinical indicators and 33 differential lipids (Red is positively correlated, green is negatively correlated, an correlation of 0.5 ~ 0.8 is moderate, a correlation of 0.3 ~ 0.5 is low ). In the volcano diagram, the horizontal coordinate represents the logarithm of the difference multiple bases 2, and a negative logarithm of the *P* -value is used as the vertical coordinate. A point's size represents its VIP value, with red representing the upregulated metabolites and blue representing the down-regulated metabolites.

On the basis of quantitative analysis, this research visualized *P* -values and multiple shift values and drew a volcanic map of the metabolites up and down to screen the differential metabolites (Fig. 3A). Using this criterion, the study selected 693 metabolites with biological significance and significant differences. A VIP value of > 1 was used in the OPLSDA model, and a *P*-value of < 0.05 was used in the analysis. Compared with the control group, there were 692 diverse lipid metabolites in the hyperuricemia group, of which 274 were upregulated, and 418 were downregulated (Fig. 3B). The study assessed 33 lipid metabolites with remarkable differences (Annex in 2 & Fig. 3C) based on FC > 2 and *P* < 0.05. According to these findings, 33 metabolites were potential markers of HUA. A correlation between clinical indicators and 33 lipids was also observed (Fig. 3D).

### Enrichment analysis of metabolic pathways in HUA patients

**Figure 4:**
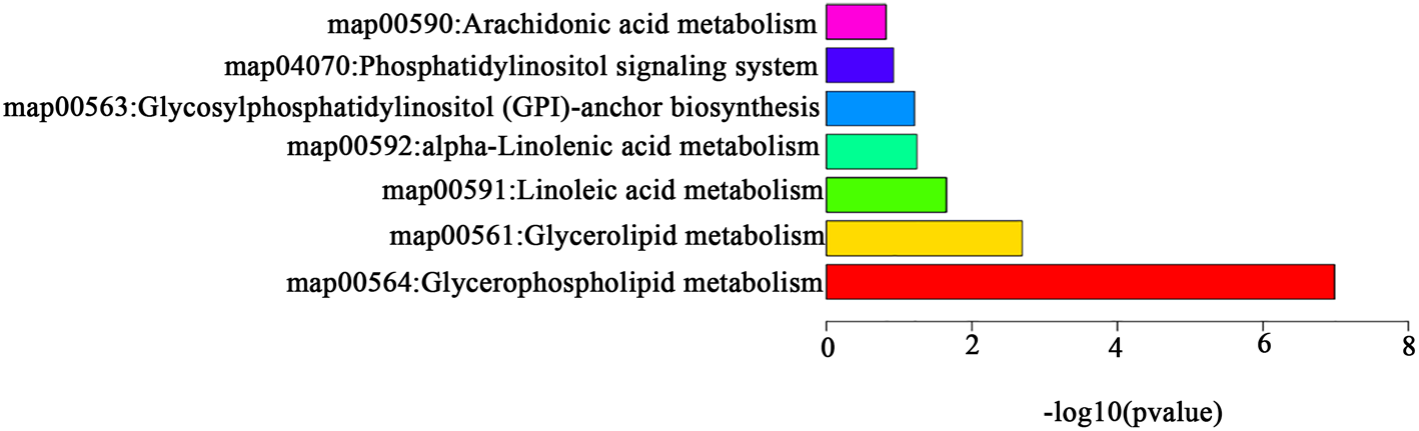
Signaling pathway weights of the primary metabolites involved.

The study query of the KEGG database yielded the metabolic pathways for 33 different metabolites (Fig. 4).

Five enriched metabolic pathways were identified as being involved in HUA's occurrence and development, including linoleic acid metabolism, glycerophospholipid metabolism, glycosylphosphatidylinositol (GPI)-anchor biosynthesis, arachidonic acid metabolism, and alpha-linolenic acid metabolism.

### Immune system results

As shown in Table 3, CPT1, TGF-β1, and LD proteins were significantly expressed in Han patients with hyperuricemia compared with Uyghur patients, whereas Glu protein levels were low. Compared with Uyghur healthy persons, CPT1 and LD proteins were highly expressed and TGF-β1, Glu and IL-6 proteins were lower in Han healthy persons (Table 4). The levels of SEP1, IL-6, TGF-β1, Glu and LD in Han and Uyghur patients were higher than those in healthy Han and Uyghur persons, while LD levels were lower (Tables 5 and 6).

**Table 3:** Analysis of the content of each index in the Han patients with hyperuricemia vs the Uyghur patients with hyperuricemia $\left( {\overline{x} \pm s} \right)$.

Group	The Han patients with hyperuricemia (n = 46)	The Uyghur patients with hyperuricemia (n = 46)	*T*-value	*P* -value
CPT1 (ng/mL)	56.884 ± 23.827 **	38.798 ± 31.289	3.119	0.002
TGF-β1 (pg/mL)	14.140 ± 3.106 **	10.845 ± 5.825	3.385	0.001
Glu (mmol/L)	4.711 ± 1.662 **	3.620 ± 1.796	− 5.107	< 0.001
LD (mmol/L)	4.933 ± 1.251 **	3.738 ± 2.255	3.143	0.002

* *P* < 0.05, ** *P* < 0.001.

**Table 4:** Analysis of the content of each index of the Han healthy persons vs the Uyghur healthy persons $\left( {\overline{x} \pm s} \right)$.

Group	The Han healthy persons (n = 30)	The Uyghur healthy persons (n = 30)	*T*-test	*P*-value
CPT1 (ng/mL)	65.307 ± 36.808**	25.449 ± 17.905	5.333	< 0.001
IL-6 (pg/mL)	1.543 ± 1.519**	1.504 ± 0.788	− 2.398	0.016
TGF-β1 (pg/mL)	12.653 ± 1.269**	8.297 ± 3.563	− 5.192	< 0.001
Glu (mmol/L)	2.881 ± 0.333**	2.014 ± 2.444	− 3.726	< 0.001
LD (mmol/L)	8.883 ± 1.184**	7.651 ± 1.238	3.939	< 0.001

* *P* < 0.05, ** *P* < 0.001.

**Table 5:** Analysis of the content of each index in the Han healthy persons VS patients with hyperuricemia $\left( {\overline{x} \pm s} \right)$.

Group	The Han healthyl persons (n = 30)	The Han patients with hyperuricemia (n = 46)	T-value	*P*-value
SEP1(μg/ml)	9.412 ± 3.826	2.097 ± 2.853 **	− 6.961	< 0.001
IL-6(pg/ml)	1.543 ± 1.519	2.643 ± 1.097 **	− 5.476	< 0.001
TGF-β1(pg/ml)	12.653 ± 1.269	14.140 ± 3.106*	− 2.898	0.005
Glu(mmol/L)	2.881 ± 0.333	4.711 ± 1.662 **	− 7.248	< 0.001
LD(mmol/L)	8.883 ± 1.184	4.933 ± 1.251 **	13.735	< 0.001

* *P* < 0.05, ** *P* < 0.001.

**Table 6:** Analysis of the content of each index in the Uyghur healthy persons VS patients with hyperuricemia $\left( {\overline{x} \pm s} \right)$.

Group	The Uyghur healthy persons (n = 30)	The Uyghur patients with hyperuricemia (n = 46)	*T*-value	*P* -value
SEP1(μg/ml)	8.015 ± 3.636	1.929 ± 1.010 **	− 6.759	< 0.001
IL-6(pg/ml)	1.504 ± 0.788	2.991 ± 2.277 **	− 6.564	< 0.001
TGF-β1(pg/ml)	8.297 ± 3.563	10.845 ± 5.825*	− 2.365	0.021
Glu(mmol/L)	2.014 ± 2.444	3.620 ± 1.796 **	− 4.448	< 0.001
LD(mmol/L)	7.651 ± 1.238	3.738 ± 2.255 **	8.677	< 0.001

* *P* < 0.05, ** *P* < 0.001.

## Discussion

The metabolic syndrome, hyperlipidemia, diabetes, and nephropathy can be linked to disorders of uric acid metabolism. In addition, these diseases can possibility increase blood uric acid levels, which can create a vicious cycle in the body. Due to body weight and BMI increases, elevated uric acid levels may be closely linked to obesity and insulin resistance in the research. As can be seen from Table 1, BMI, TG, TC, LDL-C, HDLC, VLDLC, BUN, Scr, SUA, and SUA/Scr levels in patients with hyperuricemia were all upper than in the healthy population. The increase in TG level and decrease in HDL-C level were particularly significant, indicating that dyslipidemia was common in hyperuricemia, Ning Liu et al.^19^ reported similar results_._ Confounding factor analysis in the Table 2 showed that FBG, BUN, Scr, SUA/Scr migh affected the Uric Acid Levels. The research by Alessandro M^20^ et al. showed a strong correlation between SUA and triglyceride values (the strongest correlation with triglycerides) for lipid indexes and adipose indexes. Elevated uric acid triggers an inflammatory response in the body, causing oxidative stress and promoting excessive fat metabolism in the liver, thus allowing the accumulation of free fatty acids ^6^, further indicating that elevated uric acid levels and lipid metabolism have a close relationship.

In contrast to healthy persons, the study identified nine different lipids in HUA patients by analyzing non-targeted lipid metabolism, including fatty acid (FA), phosphatidylethanolamine (PE), phosphatidylinositol (PI), phosphatidylcholine (PC), ceramide (Cer), triglyceride (TG), diacylglycerol (DG), phosphatidylserine (PS), and lysophosphatidylinositol (LPI). It was worth noting that 33 differential lipid molecules were significantly upgraded in HUA patients identified through heatmaps, among which were FA(20:4), PE[(17:0_22:6)/(16:0_18:3), OAHFA(45:6), DG[(18:2_18:2)/(38:0e)/(38:3e)/(36:1)/(36:1e)], PS(42:8), TG[(12:1e_6:0_18:3)/(16:0_18:1_24:0)/(16:0_16:0_18:2)/(16:0_18:1_22:1)/(16:2e_20:0_22:6)/(4:0_10:2_22:5)/(18:3_10:2_18:2)/(18:0_18:0_18:1)/(18:0_18:0_18:0)/(16:0_18:1_22:0)/(20:0_16:0_20:0)/(16:2e_20:2_22:6)/(20:4e_18:4_20:2)], PI(18:0_22:4), PC[(23:1_11:2)/(18:0_22:6)], PG(28:1_18:2), LPI(12:0), Hex3Cer(d34:6) and Cer[(m19:0_23:1)/(t18:0_25:3)/(m22:0_16:1)/(m18:1_22:0)]. These findings indicate that HUA patients have altered blood levels of glycerol phospholipids and their metabolites, and the disturbance of phospholipid metabolism mainly involves phosphatidylserine (PS), phosphatidylethanolamine (PE), phosphatidylcholine (PC), lysophosphatidylinositol (LPI), and phosphatidylinositol (PI). In addition to forming biofilms and recognizing cell membranes, these lipid molecules play a key role in signal transduction as well^21^. The LPCs can be activated by UA, and recombinant lyso-phosphatidylcholine acyltransferase-3 (LPCAT3) can be activated in vitro and in vivo, catalyzing PC generation^19^^,^^22^. This indicated that uric acid participates in metabolism and biosynthesis.

It is speculated that uric acid may decompose and synthesize various lipids and lipid molecules through the main lipid metabolism pathway in the human body, promoting the patient's body's lipid metabolites. Increasing the risk of HUA is increased by disordered lipid metabolism, which accelerates the accumulation of body uric acid. To further explore the influence of clinical indicators and interaction between lipids on the progression of HUA, the study used correlation analysis to analyze the interaction between clinical indicators of HUA and 33 different lipids and found that TG [(16:0_18:1_22:0)/(16:0_18:1_22:1)/(16:0_18:1_24:0)/(16:0_16:0_18:2)/20:4e_18:4_20:2)/(20:0_16:0_0)/(18:0_18:0_18:1)] and DG [(38:0e)/(36:1e)/(38:3e)] were positively related to VLDL-C and TG. CR was negatively related to TG (4:0_10:22:5), PE (16:0_18:3), PC [(18:0_22:6)/ (23:1_11:2)], and DG (36:1). BUN was negatively related to DG (18:2_18:2), LPI (12:0), PE (16:0_18:3), and TG (12:1e_6:0_18:3) levels. HUA was negatively related to DG (18:2_18:2) and TG (4:0_10:22:5) levels. HDL-C was negatively related toTG (16:0_16:0_18:2), PS (42:8), Hex 3Cer (d34:6), DG [(36:1)/(36:1e)], and Cer (m19:0_23:1). In the above study, the abnormal expression of lipid molecules were closely related to blood glucose, glomerular function, and dyslipidemia, which may explain the occurrence of hyperuricemia. These findings could be attributed to the different diets, lifestyles, and metabolism of HUA patients promoting excess nutrients and their metabolites^23^, such as free fatty acids (FFA), low-density oxidized low-density lipoprotein (OxLDL), phospholipid oxide (OxPL), ceramides (Cer), and UA. A chronic low-grade inflammation in HUA is caused by these endogenous danger signaling molecules, which activate pro-inflammatory signaling pathways and release pro-inflammatory mediators. This study's results were also related to the finding that metabolic overload induces aseptic inflammation^24^. The study speculated that different lipid molecules and lipid metabolites in the inflammatory process of HUA could function as proinflammatory mediators by stimulating various immune receptors to initiate the downstream inflammatory cascade. Furthermore, it is involved in signal transduction in lipid metabolism.

The research conducted pathway analysis using LIPEA based on KEGG database sources^25^ to study pathways related to lipid changes. The influence values of glycosylphosphatidylinositol (GPI) -anchor biosynthesis, glycerophospholipid metabolism, arachidonic acid metabolism, alpha-linolenic acid metabolism, and linoleic acid metabolism pathways in HUA patients were 0.216, 0, 0, 0.004, and 0, respectively, indicating that elevated uric acid mainly leads to changes in glycophosphatidylinositol-anchor biosynthesis and glycerophospholipid metabolism in the human body. A similar finding was observed in rats with an elevated uric acid level by Liu Mingyu et al.^26^ suggesting that excess uric acid enhances the glycosylphosphatidylinositol-anchor biosynthesis, immunity, inflammation, and autophagy-related pathways. The study speculate that the lipid metabolism of HUA could be regulated through a metabolic microenvironment composed of various vital enzymes, transcription factors, and lipids. Furthermore, abnormal lipid metabolism also shifts the metabolic signaling pathway in cells of patients with hyperuricemia and affects the normal cell population of the human body through lipid substances. This complexity indicates that it is important to examine not only the HUA lipid metabolic network, but also the pathways by which HUA interacts with lipids and the influence of cellular lipid metabolism on its progress and outcomes.

The above studies demonstrated that glycerophospholipids accounted for a considerable proportion of all the differential metabolites in patients with hyperuricemia, and the metabolic pathway of glycerophospholipid metabolism has interfered. Glycerol, fatty acids, and phospholipids are vital components of glycerophospholipids, among which dietary fatty acids can enhance fatty acid oxidation (FAO) in the mitochondria, accelerating production of TG in fat droplets, and provide more energy to liver cells^28^. Therefore, liver fat can be prevented from accumulating by oxidizing fatty acids, resulting in a lipid metabolism disorder^28^. However, inhibition of mitochondrial FAO affects the expression of key regulatory factors in the energy metabolism pathway and modifies the levels of intracellular metabolites, thus reshaping intracellular energy metabolism homeostasis^29^. CPT1 is a crucial enzyme and regulatory factor in the fatty acid β-oxidation that can regulate lipid metabolism and improve body fat content. As can be seen from Tables 3 and 4, the expression level of CPT1 in the Han population was significantly higher than that in the Uyghur population in both patients with hyperuricemia and healthy people. The study speculated that exogenous dietary intake of elevated fat and sugar levels and endogenous dyslipidemia metabolism could increase the fatty acid content in Han patients with hyperuricemia, and this leads to an excess of free fatty acids, which induces the expression of fatty acid oxidase CPT1, increasing fatty acid oxidation, and causing the lysis of lipids. Patsoukis et al.^30^ confirmed these findings, and they believed that this process mainly promoted fatty acid oxidation of endogenous lipids through the release of TGhydrolase, denutrient/fatty acid triglyceride lipase (ATGL), fatty acids, and glycerol. A study found that increased fatty acid oxidation^31^ can affect glycolysis-related cell metabolism. Glu and LD are critical intermediates in gluconeogenesis, and glucose conversion produces lactic acid to pyruvate via glycolysis and LDH. Increased oxidation of fatty acids decreases pyruvate dehydrogenase (PDH) activity, inhibits Glu glycolysis, inhibits glucose transporter (Glut-1) expression and Glu uptake, and increases glucose utilization by activated T cells. Meanwhile, It reduces intracellular glucose and 6 monophosphate glucose, while increasing glucoselytic intermediaries 1 and 2, as well as LD and pyruvate. The increase in LD levels may also symbolize the metabolic migration to glycolysis related to inflammation^33^, suggesting that LD, the final product of aerobic glycolysis, maybe the medium that causes the disorder of lipid metabolism in HUA to produce an inflammatory state. TGF-β1, a transforming glycoprotein growth factor, can participate in cell proliferation, differentiation, behavioral induction, and survival. According to research, hyperuricemia injury can increase the expression and phosphorylation of TGF-β1 in the kidney^34^. Due to uric acid stimulation, the TGF- β signaling pathway is activated^35^ and the mTOR signaling pathway is downregulated^35^, leading to increased oxidative stress, IL-6, IL-10 levels, and other inflammatory factors^36^. According to Table 3, the study's results were similar with previous ones, which found that CPT1, TGF-β1, and LD levels proteins were elevated, and Glu levels were low in Han patients with HUA. The study speculated that increased CPT1, a key substance regulating lipid metabolism in the carnitine transport system, may promote the oxidation of fatty acids, thus inhibiting glycolysis by reducing Glu and increasing LD. Thus, CPT1 may promote oxidative phosphorylation, resulting in increased levels of TGF-β1 and cellular inflammatory factors (IL-6 and IL-10), thus promoting the inflammatory state of HUA.

### Advantages and shortcomings

The advantage of this study lies in the differential metabolites identification and metabolic pathways in patients with hyperuricemia by lipid metabolomics technology, and objective analysis of possible pathogenesis of abnormal expression of immune factors in differential metabolic pathways combined with ELISA. The disadvantage is that there is a limited number of participants in this study, and further large sample detection is needed to improve the reliability and universality of the research results. Second, the validation tests in this study were too simplistic, and a comprehensive analysis of lipid metabolism in hyperuricemia by transcriptome sequencing and proteomics is necessary to determine whether these indicators contribute to predicting HUA risk, and to illuminate downstream and HUA disease progression using regulatory metabolites.

## Conclusion

This study screened for differential metabolites of HUA lipids and their related lipid molecules and identified key lipid molecules that participate in glycerophospholipid metabolism, arachidonic acid metabolism, linoleic acid metabolism, glycosylphosphatidylinositol (GPI)—anchor biosynthesis, and alpha-linolenic acid metabolism pathways. Furthermore, the study identified that the glycerophospholipid metabolic pathway is crucial for the progression of HUA. By detecting changes in the expression levels of key regulatory factors in the metabolic pathway, we speculated that the regulatory factors CPT1, TGF-β1, Glu, and LD cytokines could promote fatty acid oxidation, inhibit glycolysis, and promote oxidative phosphorylation metabolism through the glycerophospholipid metabolic pathway of glycerophospholipids, and then regulate and affect metabolism microenvironment in patients with hyperuricemia after reprogramming. This may be a target for future intervention or a key point for further studies of HUA. Different metabolite markers can provide evidence linking lipid metabolism disorders with HUA at the molecular level and provide hypotheses for future research. This study suggests that regular monitoring of lipid indexes in patients with hyperuricemia should be conducted in clinical practice, and lipid-lowering drug intervention is necessary for patients with hyperuricemia with obvious dyslipidemia, which may delay the development of HUA to a certain extent.

### Supplementary Information

Supplementary Information 1.

Supplementary Information 2.

Supplementary Information 3.

Supplementary Information 4.

Supplementary Information 5.

## Data Availability

You can view all primary data presented in the study in the article/supplementary materials.Please contact Lili Ma if someone wants to request data from this study. All data generated or analysed during this study are included in this published article (and its Supplementary Information files).

## Abbreviations

HPLC: High-performance liquid chromatography
LC-MS: Liquid chromatography-mass spectrometry
BMI: Body mass index,
UA: Uric acid
FBG: Fasting blood glucose
TC: Total cholesterol
TG: Triglycerides
HDL-C: High-density lipoprotein cholesterol
LDL-C: Low-density lipoprotein cholesterol
VLDL-c: Very low-density lipoprotein cholesterol
BUN: Blood urea nitrogen
CR: Creatinine
CPT1: Carnitine palmitoyltransferase-1
SEP1: Human selenoprotein 1
IL-6: Interleukin 6
IL-10: Interleukin 10
TNF-α: Tumor necrosis factor-α
TGF-β1: Transforming growth factor-β1
SEP1: Selenoprotein 1
Glu: Glucose
LD: Lactic acid
PE: Phosphatidylethanolamine
PI: Phosphatidylinositol
PC: Phosphatidylcholine
Cer: Ceramide
DG: Diacylglycerol
PS: Phosphatidylserine
LPI: Lysophosphatidylinositol
