# Supplementary material for: The effect of lipid metabolism disorder on patients with hyperuricemia using Multi-Omics analysis
Source: Sci Rep. 2023 Oct 24;13:18211. doi: 10.1038/s41598-023-45564-8 (PMC10598229; doi:10.1038/s41598-023-45564-8)
Supplement: Supplementary file 4 — Supplementary Information 4. [file 41598_2023_45564_MOESM4_ESM.doc]

| **Supplementary table 1.** **Correlation analysis between variables and SUA** | | |
| --- | --- | --- |
| **Variable** | r | ***P* -value** |
| **Age(years)** | 0.117 | 0.205 |
| **Sex** | -0.749** | 0.000 |
| **Ethnicity** | 0.002 | 0.981 |
| **Weight (kg)** | 0.528** | 0.000 |
| BMI**(kg/m2)** | 0.284** | 0.002 |
| TC**(mmol/L)** | 0.258** | 0.004 |
| TG**(mmol/L)** | 0.632** | 0.000 |
| HDLC**(mmol/L)** | -0.661** | 0.000 |
| LDLC**(mmol/L)** | 0.467** | 0.000 |
| VLDLC**(mmol/L)** | 0.281** | 0.002 |
| **FBG(mmol/L)** | -0.086 | 0.349 |
| BUN**(mmol/L)** | 0.314** | 0.000 |
| Scr**(mmol/L)** | 0.511** | 0.000 |
| SUA/Scr**(mmol/L)** | 0.803** | 0.000 |
| Cardiovascular event | 0.375** | 0.000 |
