# Supplementary material for: The effect of lipid metabolism disorder on patients with hyperuricemia using Multi-Omics analysis
Source: Sci Rep. 2023 Oct 24;13:18211. doi: 10.1038/s41598-023-45564-8 (PMC10598229; doi:10.1038/s41598-023-45564-8)
Supplement: Supplementary file 5 — Supplementary Information 5. [file 41598_2023_45564_MOESM5_ESM.doc]

| Variable in an equation | | | | | | | | | |
| --- | --- | --- | --- | --- | --- | --- | --- | --- | --- |
|  | | B | Standard error | Wald | Degree of freedom | Significance | Exp(B) | 95% confidence interval for EXP(B) | |
|  |  |  |  |  |  |  |  | floor | Upper |
| step 1a | Uric acid to creatinine ratio | 2.629 | .459 | 32.879 | 1 | .000 | 13.867 | 5.645 | 34.065 |
|  | constant | -12.350 | 2.143 | 33.225 | 1 | .000 | .000 |  |  |
| a. Variable entered in step 1: uric acid to creatinine ratio. | | | | | | | | | |

d
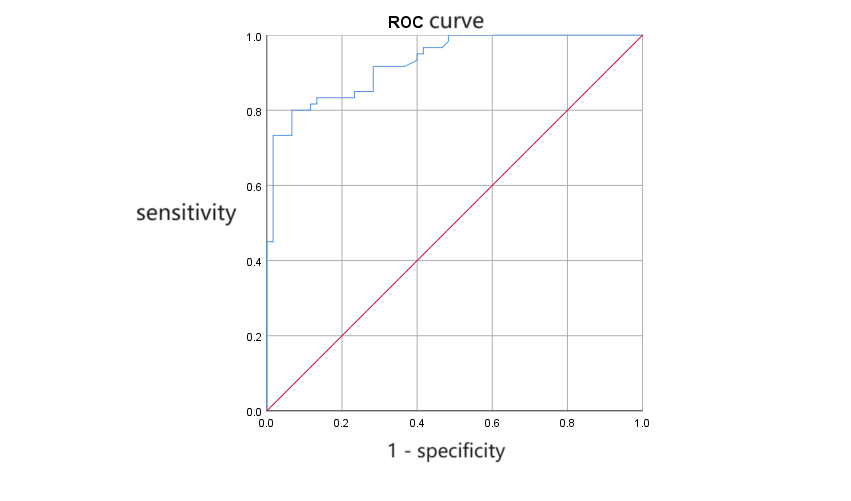


| The area below the curve | | | | |
| --- | --- | --- | --- | --- |
| Test result variable: ratio of uric acid to creatinine | | | | |
| Region | Standard errora | Asymptotic significanceb | Asymptotic 95% confidence interval | |
|  |  |  | floor | Upper |
| .928 | .022 | .000 | .885 | .971 |

| Coordinates of a curve |  |  |  |  |  |  |
| --- | --- | --- | --- | --- | --- | --- |
| Test result variable: ratio of uric acid to creatinine |  |  |  |  |  |  |
| Greater than or equal to this value is positive a | Sensitivity | 1-specificity | Specificity | Jorden index |  |  |
| 1.49 | 1 | 1 | 0 | 0 |  |  |
| 2.585 | 1 | 0.983 | 0.017 | 0.017 |  |  |
| 2.705 | 1 | 0.967 | 0.033 | 0.033 |  |  |
| 2.845 | 1 | 0.95 | 0.05 | 0.05 |  |  |
| 2.97 | 1 | 0.933 | 0.067 | 0.067 |  |  |
| 2.985 | 1 | 0.917 | 0.083 | 0.083 |  | max |
| 3.005 | 1 | 0.9 | 0.1 | 0.1 |  | 0.733 |
| 3.065 | 1 | 0.883 | 0.117 | 0.117 |  |  |
| 3.17 | 1 | 0.867 | 0.133 | 0.133 |  |  |
| 3.26 | 1 | 0.85 | 0.15 | 0.15 |  |  |
| 3.315 | 1 | 0.833 | 0.167 | 0.167 |  |  |
| 3.365 | 1 | 0.817 | 0.183 | 0.183 |  |  |
| 3.425 | 1 | 0.8 | 0.2 | 0.2 |  |  |
| 3.485 | 1 | 0.783 | 0.217 | 0.217 |  |  |
| 3.535 | 1 | 0.75 | 0.25 | 0.25 |  |  |
| 3.595 | 1 | 0.733 | 0.267 | 0.267 |  |  |
| 3.64 | 1 | 0.717 | 0.283 | 0.283 |  |  |
| 3.655 | 1 | 0.7 | 0.3 | 0.3 |  |  |
| 3.665 | 1 | 0.683 | 0.317 | 0.317 |  |  |
| 3.68 | 1 | 0.667 | 0.333 | 0.333 |  |  |
| 3.73 | 1 | 0.65 | 0.35 | 0.35 |  |  |
| 3.785 | 1 | 0.633 | 0.367 | 0.367 |  |  |
| 3.805 | 1 | 0.617 | 0.383 | 0.383 |  |  |
| 3.815 | 1 | 0.583 | 0.417 | 0.417 |  |  |
| 3.825 | 1 | 0.567 | 0.433 | 0.433 |  |  |
| 3.84 | 1 | 0.55 | 0.45 | 0.45 |  |  |
| 3.875 | 1 | 0.517 | 0.483 | 0.483 |  |  |
| 3.925 | 1 | 0.5 | 0.5 | 0.5 |  |  |
| 3.965 | 1 | 0.483 | 0.517 | 0.517 |  |  |
| 3.985 | 0.983 | 0.483 | 0.517 | 0.5 |  |  |
| 3.995 | 0.967 | 0.467 | 0.533 | 0.5 |  |  |
| 4.02 | 0.967 | 0.45 | 0.55 | 0.517 |  |  |
| 4.05 | 0.967 | 0.433 | 0.567 | 0.534 |  |  |
| 4.065 | 0.967 | 0.417 | 0.583 | 0.55 |  |  |
| 4.075 | 0.95 | 0.417 | 0.583 | 0.533 |  |  |
| 4.09 | 0.95 | 0.4 | 0.6 | 0.55 |  |  |
| 4.11 | 0.933 | 0.4 | 0.6 | 0.533 |  |  |
| 4.16 | 0.917 | 0.367 | 0.633 | 0.55 |  |  |
| 4.205 | 0.917 | 0.35 | 0.65 | 0.567 |  |  |
| 4.215 | 0.917 | 0.333 | 0.667 | 0.584 |  |  |
| 4.245 | 0.917 | 0.317 | 0.683 | 0.6 |  |  |
| 4.28 | 0.917 | 0.3 | 0.7 | 0.617 |  |  |
| 4.295 | 0.917 | 0.283 | 0.717 | 0.634 |  |  |
| 4.315 | 0.9 | 0.283 | 0.717 | 0.617 |  |  |
| 4.335 | 0.883 | 0.283 | 0.717 | 0.6 |  |  |
| 4.35 | 0.867 | 0.283 | 0.717 | 0.584 |  |  |
| 4.365 | 0.85 | 0.283 | 0.717 | 0.567 |  |  |
| 4.385 | 0.85 | 0.267 | 0.733 | 0.583 |  |  |
| 4.415 | 0.85 | 0.25 | 0.75 | 0.6 |  |  |
| 4.45 | 0.85 | 0.233 | 0.767 | 0.617 |  |  |
| 4.48 | 0.833 | 0.233 | 0.767 | 0.6 |  |  |
| 4.51 | 0.833 | 0.2 | 0.8 | 0.633 |  |  |
| 4.545 | 0.833 | 0.183 | 0.817 | 0.65 |  |  |
| 4.605 | 0.833 | 0.167 | 0.833 | 0.666 |  |  |
| 4.68 | 0.833 | 0.15 | 0.85 | 0.683 |  |  |
| 4.715 | 0.833 | 0.133 | 0.867 | 0.7 |  |  |
| 4.755 | 0.817 | 0.133 | 0.867 | 0.684 |  |  |
| 4.795 | 0.817 | 0.117 | 0.883 | 0.7 |  |  |
| 4.815 | 0.8 | 0.117 | 0.883 | 0.683 |  |  |
| 4.86 | 0.8 | 0.1 | 0.9 | 0.7 |  |  |
| 4.895 | 0.8 | 0.083 | 0.917 | 0.717 |  |  |
| 4.945 | 0.8 | 0.067 | 0.933 | 0.733 |  |  |
| 5.01 | 0.767 | 0.067 | 0.933 | 0.7 |  |  |
| 5.05 | 0.733 | 0.067 | 0.933 | 0.666 |  |  |
| 5.075 | 0.733 | 0.05 | 0.95 | 0.683 |  |  |
| 5.085 | 0.733 | 0.033 | 0.967 | 0.7 |  |  |
| 5.105 | 0.733 | 0.017 | 0.983 | 0.716 |  |  |
| 5.135 | 0.717 | 0.017 | 0.983 | 0.7 |  |  |
| 5.205 | 0.7 | 0.017 | 0.983 | 0.683 |  |  |
| 5.265 | 0.683 | 0.017 | 0.983 | 0.666 |  |  |
| 5.29 | 0.667 | 0.017 | 0.983 | 0.65 |  |  |
| 5.315 | 0.65 | 0.017 | 0.983 | 0.633 |  |  |
| 5.325 | 0.633 | 0.017 | 0.983 | 0.616 |  |  |
| 5.37 | 0.617 | 0.017 | 0.983 | 0.6 |  |  |
| 5.415 | 0.583 | 0.017 | 0.983 | 0.566 |  |  |
| 5.445 | 0.567 | 0.017 | 0.983 | 0.55 |  |  |
| 5.475 | 0.55 | 0.017 | 0.983 | 0.533 |  |  |
| 5.495 | 0.533 | 0.017 | 0.983 | 0.516 |  |  |
| 5.525 | 0.517 | 0.017 | 0.983 | 0.5 |  |  |
| 5.545 | 0.5 | 0.017 | 0.983 | 0.483 |  |  |
| 5.595 | 0.483 | 0.017 | 0.983 | 0.466 |  |  |
| 5.695 | 0.467 | 0.017 | 0.983 | 0.45 |  |  |
| 5.76 | 0.45 | 0.017 | 0.983 | 0.433 |  |  |
| 5.775 | 0.45 | 0 | 1 | 0.45 |  |  |
| 5.79 | 0.433 | 0 | 1 | 0.433 |  |  |
| 5.81 | 0.417 | 0 | 1 | 0.417 |  |  |
| 5.83 | 0.4 | 0 | 1 | 0.4 |  |  |
| 5.855 | 0.383 | 0 | 1 | 0.383 |  |  |
| 5.9 | 0.367 | 0 | 1 | 0.367 |  |  |
| 5.935 | 0.35 | 0 | 1 | 0.35 |  |  |
| 5.955 | 0.333 | 0 | 1 | 0.333 |  |  |
| 5.975 | 0.317 | 0 | 1 | 0.317 |  |  |
| 6.005 | 0.3 | 0 | 1 | 0.3 |  |  |
| 6.055 | 0.283 | 0 | 1 | 0.283 |  |  |
| 6.135 | 0.25 | 0 | 1 | 0.25 |  |  |
| 6.205 | 0.233 | 0 | 1 | 0.233 |  |  |
| 6.25 | 0.217 | 0 | 1 | 0.217 |  |  |
| 6.315 | 0.2 | 0 | 1 | 0.2 |  |  |
| 6.425 | 0.183 | 0 | 1 | 0.183 |  |  |
| 6.535 | 0.167 | 0 | 1 | 0.167 |  |  |
| 6.575 | 0.15 | 0 | 1 | 0.15 |  |  |
| 6.61 | 0.133 | 0 | 1 | 0.133 |  |  |
| 6.665 | 0.117 | 0 | 1 | 0.117 |  |  |
| 6.695 | 0.1 | 0 | 1 | 0.1 |  |  |
| 6.85 | 0.083 | 0 | 1 | 0.083 |  |  |
| 7.06 | 0.067 | 0 | 1 | 0.067 |  |  |
| 7.165 | 0.05 | 0 | 1 | 0.05 |  |  |
| 7.315 | 0.033 | 0 | 1 | 0.033 |  |  |
| 7.61 | 0.017 | 0 | 1 | 0.017 |  |  |
| 8.8 | 0 | 0 | 1 | 0 |  |  |
